# Assessment of prevalence, predictors, reasons and regulations of substance smoking among children in Ghana

**DOI:** 10.1186/s12889-023-17187-1

**Published:** 2023-11-16

**Authors:** Sylvester Kyei-Gyamfi, Frank Kyei-Arthur

**Affiliations:** 1 Department of Children, Ministry of Gender, Children and Social Protection, Accra, Ghana; 2 Department of Environment and Public Health, University of Environment and Sustainable Development, Somanya, Ghana

**Keywords:** Smoking, Children, Shisha, Cigarettes, Regulations, Marijuana, Tobacco, Ghana

## Abstract

**Background:**

In Ghana, it is against the law for children to smoke. Nevertheless, a portion of children in the country do smoke. However, there is a paucity of research on young smokers in Ghana and other sub-Saharan African nations. This study, therefore, investigated the prevalence of smoking, the kinds of substances children smoke, predictors of smoking, reasons for and factors that lead children to smoke, and regulation of smoking among children in Ghana.

**Methods:**

In total, 5024 children aged 8–17 were studied using a cross-sectional convergent parallel mixed method. Descriptive statistics, Person’s Chi-square test, Fisher Exact test, and binary logistic regression were used to analyse the quantitative data. In contrast, thematic analysis was used to analyse the qualitative data.

**Results:**

The results showed that 3.2% of children had ever smoked. Male children smoked more often than female children. The prevalence of cigarette, marijuana, and shisha smoking was 78.3%, 18%, and 3.7%, respectively. While more male children than female children smoked cigarettes and marijuana, more female children than male children used shisha. Children mainly smoked for fun and due to group culture. In addition, children were influenced by friends; parents, family members, and neighbours who smoke; curiosity; and advertisements and videos to initiate smoking. Despite the availability of regulations and laws regulating tobacco products in public places, tobacco advertisement, promotion, and sale to persons under 18, children are still smoking.

**Conclusions:**

Children who have ever engaged in smoking reported using cigarettes, marijuana, and shisha. Policymakers urgently need to strategise and strengthen their policies, programmes, and interventions to address smoking among children.

**Supplementary Information:**

The online version contains supplementary material available at 10.1186/s12889-023-17187-1.

## Background

Smoking among people under 18 is increasingly becoming a public health concern in Ghana. The laws of Ghana prohibit smoking among persons under 18, yet there is evidence of children smoking. The Tobacco Control Regulations 2016 (L. I. 2247) and the Public Health Act 2012 (Act 851) are some laws that seek to prohibit and regulate the sale of tobacco substances to persons under 18. Despite these laws, the 2016 Multiple Indicator Cluster Survey (MICS) [1] indicates that 1% of adolescent girls and 6% of adolescent boys aged 15–19 have ever used tobacco. The Ministry of Gender, Children and Social Protection (MoGCSP) also reports that some children in Senior High Schools smoke marijuana, and about 90% of cases at the Accra Psychiatric Hospital annually, are drug abuse related among young people [2].

In Ghana, studies have documented that children engage in shisha/waterpipe, cigarette, and marijuana smoking [3–5]. For instance, Asante et al.‘s [4] study found that the prevalence of the use of marijuana among young people aged 11–19 years was 5.3%. Logo et al.‘s [5] study using nationally representative data on Junior High School students in Ghana found that 3.1% of students had ever smoked shisha/waterpipe. In comparison, Amonoo-Lartson and Pappoe’s [3] study among students in Senior High School found that the prevalence of smoking only cigarettes, only marijuana, and both cigarettes and marijuana were 5.3%, 0.6%, and 5.2%, respectively.

Although the prevalence of tobacco use among children in Ghana is low, it is still worrying since it is illegal and has dire consequences for their safety and health. Studies have linked substance smoking to an increased risk of premature deaths, disabilities, bad breath, respiratory diseases, sleep-related impairments, and non-communicable diseases among smokers [6–9].

Since early smoking initiation negatively impacts health, measures to reduce smoking among children and adolescents are vital [10]. However, a few nationally representative studies have been conducted on smoking among children in Ghana [1, 4, 5, 11]. Effective smoking prevention measures require an in-depth understanding of children’s smoking experiences. The current study, which used nationally representative data, is of utmost significance since it will enhance policymakers’ understanding of children’s smoking experiences to further strategise national programmes, policies, and interventions to reduce its prevalence among children. The study aimed to investigate the prevalence of smoking, the kinds of substances children smoke, and predictors of smoking in Ghana. It also examined the reasons for and factors that lead children to smoke and the regulation of smoking among children in Ghana.

## Methods

### Study design and sampling procedure

This study used a convergent parallel mixed-method design. A convergent parallel mixed-method design is a type of mixed-method in which the researcher concomitantly collects quantitative and qualitative data to compare, relate or validate findings [12]. The researchers in this study used a multi-stage sampling strategy. Twenty percent (20%) of districts throughout all ten regions of Ghana were randomly selected based on child protection criteria. Of 216 districts across the country, 43 districts were randomly selected. In each of the 43 districts, 15 enumeration areas (EAs) were randomly selected for enumeration. In total, 645 EAs were randomly selected. Third, 8 children aged 8–17 in households were randomly selected in each EA. In households with two or more children aged 8–17, the youngest child was selected. In total, 5,160 children aged 8–17 were eligible to be interviewed. Out of the 5,160 children aged 8–17 who were eligible to be interviewed, 5,024 children were randomly selected to participate in the study. In total, 5,024 children aged 8–17 were interviewed using a semi-structured questionnaire for the quantitative data.

For the qualitative data collection, a focus group discussion (FGD) was conducted with children aged 8–17 in each of Ghana’s ten regions to explore children’s substance smoking behaviours, including the types of substances children smoke, the reasons why children smoke, and locations where children smoke. The FGDs were conducted in Akwamufie, Nkran*-*Nkwanta, Elmina, Atimpoku, Agyei-Kojo, Yendi, Gongnia, Jirapa, Nkonya, and Huni-Valley. In total, ten FGDs were conducted with children aged 8–17, and the discussions were conducted in town halls in the ten communities. Each FGD had between 8 and 10 participants. In total, 92 participants participated in the ten FGDs. In terms of age, 32 participants were aged 14–17, while 30 participants each were aged 8–10 and 11–13. Three trained research assistants conducted the FGDs in each community. One trained research assistant was the moderator, while the remaining two trained research assistants were the notetakers. All FGDs were audio-recorded. The FGDs were conducted in English and local languages convenient for participants. The local languages included Twi, Fante, Ga, Ewe, Kasem, Gruni, and Dagbani.

In addition, 50 key informant interviews were conducted with staff of selected District Assemblies and state agencies that regulate substance use in the country and those that protect and promote the wellbeing of children. The staff of state agencies interviewed included the Department of Children (DOC), MoGCSP; Narcotics Control Board (NACOB); and Food and Drugs Authority (FDA).

### Study setting

Ghana is located in West Africa, and it is bounded by Burkina Faso to the North, the Gulf of Guinea to the South, Togo to the East, and Côte d’Ivoire to the West. During the data collection in April-September 2018, Ghana had ten regions. However, following a referendum in December 2018, Ghana now has 16 regions. According to the Ghana Statistical Service, 30.8 people were residing in Ghana as of 2021 [13].

### Description and conceptualisation of variables

#### Dependent variable

The dependent variable for this study was whether children had ever smoked any substance, and the responses were Yes and No. Previous studies on smoking [3, 14] guided the development of questions on smoking in the questionnaire (See Section L 12–16 of the supplementary file).

#### Independent variables

The independent variables for this study included sex (male and female), age (8–10 years, 11–13 years, and 14–17 years), educational attainment (not in school, primary, Junior High School (JHS), Senior High School (SHS) and Tertiary), religion (Christianity, Islam, and other), ecological zone (Coastal, Middle belt, and Northern zones), living arrangements (Both Biological Parents, Biological Mother Alone, Biological Father Alone, and Other), and currently working (Yes and No).

### Data collection

This research is a subset of a larger project analysing the living conditions of children in Ghana. The larger project covered varied topics, including children’s rights, access to quality healthcare, sexual and reproductive health education, and internet and social media use (See supplementary file). This study focused on smoking among children. The data were collected between April and September 2018. The quantitative data were collected from children aged 8–17, while the qualitative data were collected from key informants and children aged 8–17.

Participation in the study was voluntary; all participants gave their written consent before their data were collected. For children aged 8–17, their parents and guardians gave their written consent before they were invited to participate in the study. The study was approved by the Institutional Review Board of the Department of Children, Ministry of Gender, Children and Social Protection (MoGCSP). The principles of the Helsinki Declaration were also followed during the data collection, and participants’ data were anonymised to protect their privacy.

### Data analysis

The Statistical Package for Social Sciences (SPSS) version 26 was used to analyse the quantitative data. Descriptive statistics, such as frequencies and percentages, were used to describe the socio-demographic characteristics of participants and the prevalence of ever smoking. Associations between ever smoking and socio-demographic characteristics (sex, age, educational attainment, religion, ecological zone, living arrangement, and currently working) were examined using Person’s Chi-square test. Also, Fisher Exact test was used to test the association between ever smoking and educational attainment since a category of educational attainment had a cell count of less than 5. Binary logistic regression was used to determine predictors of smoking among children. Binary logistic regression was used because the dependent variable (whether children had ever smoked) had dichotomous outcomes (Yes and No). All associations between variables are statistically significant at a 95% confidence interval (P < 0.05).

The qualitative data was analysed thematically. All participants’ transcripts were read to get a general sense of the smoking experiences of children aged 8–17. Perceptions of participants that aligned with the objectives of the study were assigned unique codes. Similar unique codes were merged into sub-themes. Sub-themes related to similar issues were integrated into themes.

## Results

### Socio-demographic characteristics of participants

The socio-demographic characteristics of the 5,024 sampled children aged 8–17 are presented in Table 1. According to Table 1, a little over half (51.1%) of the children interviewed were males. Most children (75.1%) were Christians, while 21.6% were Muslims. The age breakdown of the children shows that a larger proportion (43.7%) were between the ages of 14 and 17.

**Table 1:** Socio-demographic characteristics of participants

Variables	Frequency	Percent
*Sex*		
Male	2566	51.1
Female	2458	48.9
*Age*		
8–10	1330	26.5
11–13	1497	29.8
14–17	2197	43.7
Mean ± Standard deviation	12.9 ± 3.0	
*Educational attainment*		
No education	27	0.5
Primary	2116	42.1
JHS	1584	31.5
SHS	1183	23.5
Tertiary	114	2.3
*Religion*		
Christianity	3771	75.1
Islam	1083	21.6
Other	170	3.4
*Ecological zone*		
Coastal zone	1806	35.9
Middle belt	2043	40.7
Northern zone	1175	23.4
*Living arrangements*		
Both biological parents	2883	58.4
Biological mother alone	987	19.6
Biological father alone	225	4.5
Other	929	18.5
*Currently working*		
Yes	200	4.0
No	4824	96.0

In terms of education, 42.1% were in primary school, 31.5% were in JHS, 23.5% were in SHS, a little over 2% were in tertiary institutions, and less than 1% had no formal education. The number of children sampled was highest in the Middle belt (40.7%) and lowest in the Northern zone (23.4%). Regarding living arrangements, most children (58.4%) lived with their biological parents, while about 5% lived with their biological father. The majority of children (96%) were not currently working.

### Prevalence of ever smoked

In this study, 3.2% of the children sampled reported they had ever smoked, with more males (5.5%) than females (0.8%) doing so (Table 2). The percentage of children who had ever smoked was significantly associated with age (p-value = < 0.001), with children aged 14–17 having the highest rate, followed by those aged 11–13 (1.0%) and 8–10 (0.8%). Also, participants’ educational attainment was significantly associated with children ever smoked (p-value = < 0.001), with the reported smoking habit being highest among SHS children and least among children who had no formal education.

**Table 2:** Children who had ever smoked by sex, age, education, religion, and region

Variables	Have you ever smoked?		P-values
	Yes (%)	No (%)	
*Sex*			< 0.001
Male	142 (5.5)	2424 (94.5)	
Female	19 (0.8)	2439 (99.2)	
*Age*			< 0.001
8–10	10 (0.8)	1320 (99.2)	
11–13	15 (1.0)	1482 (99.0)	
14–17	136 (6.2)	2061 (93.8)	
*Educational attainment*			< 0.001*
No Education	0 (0.0)	27 (100.0)	
Primary	19 (0.9)	2097 (99.1)	
JHS	36 (2.3)	1548 (97.7)	
SHS	88 (7.4)	1095 (92.6)	
Tertiary	181 (15.8)	96 (84.2)	
*Religion*			< 0.001
Christianity	51 (14.2)	309 (85.8)	
Islam	21 (4.4)	453 (95.6)	
Other	19 (5.0)	358 (95.0)	
*Ecological zone*			< 0.001
Coastal zone	103 (5.7)	1703 (94.3)	
Middle belt	45 (2.2)	1998 (97.8)	
Northern zone	13 (1.1)	1162 (98.9)	
*Living arrangements*			< 0.001
Both biological parents	76 (2.6)	2807 (97.4)	
Biological mother alone	29 (2.9)	958 (97.1)	
Biological father alone	17 (7.6)	220 (92.4)	
Other	39 (4.2)	890 (95.8)	
*Currently working*			< 0.001
Yes	24 (12.0)	176 (88.0)	
No	137 (2.8)	4687 (97.2)	
Total	161 (3.2)	4863 (96.8)	

* Fisher Exact test

The highest prevalence of ever smoked was found among children who were Christians (14.2%), belonged to other religious categories (5.0%), and Muslims (4.4%). The association between children ever smoked, and religion was statistically significant (p-value = < 0.001). Children in the Coastal zone had the highest proportion (5.7%) of children who had ever smoked, while children from the Northern zone had the lowest proportion (1.1%). The association between children ever smoked and the ecological zone was statistically significant (p-value = < 0.001).

Furthermore, the percentage of children who had ever smoked was significantly associated with living arrangements (p-value = < 0.001), with children living with their biological father having the highest proportion (7.6%) of children who had ever smoked, while children living with their biological parents having the lowest proportion (2.6%). The highest prevalence of ever smoking was found among children currently working (12.0%). The association between children ever smoked and children currently working was statistically significant (p-value = < 0.001).

### Kinds of substances children smoked

Table 3 presents the kind of substances children smoked. The results show that nearly eight out of ten children (78.3%) smoked cigarettes, 18% smoked marijuana, and 3.7% smoked shisha. Also, a higher proportion of male children smoked cigarettes (78.9%) and marijuana (18.3%) than female children (73.7% and 15.8%). However, more female children (10.5%) smoked shisha than male children (2.8%). In terms of frequency of smoking, most children (57.1%) smoked once, followed by those who smoked several times (32.3%). A higher proportion of male children (58.5%) smoked once than female children (47.4%), while a higher proportion of female children (36.8%) smoked several times than male children (31.7%).

**Table 3:** Kind of substance children smoked and number of times children have ever smoked by sex of children

Response	Male (%)	Female (%)	Total (%)
*What children smoke*			
Cigarette	112 (78.9)	14 (73.7)	126 (78.3)
Marijuana	26 (18.3)	3 (15.8)	29 (18.0)
Shisha	4 (2.8)	2 (10.5)	6 (3.7)
*Number of times smoked*			
Once	83 (58.5)	9 (47.4)	92 (57.1)
Twice	7 (4.9)	1 (5.3)	8 (5.0)
Several times	45 (31.7)	7 (36.8)	52 (32.3)
Cannot recall	7 (4.9)	2 (10.5)	9 (5.6)

### Smoking of cigarette

The FGDs provided information on the kind of illicit substances children typically smoke. Most interactions in rural and urban settings demonstrated that cigarettes are the most popular tobacco product smoked by children in the country. Children frequently purchase cigarettes from adult peddlers in urban areas because they cannot purchase them from stores. A child reported that:

*Most of the younger people who smoke in [Tema] Community 3, where I reside, are boys, and all smoke cigarettes. Children in Tema Township find it very challenging to purchase cigarettes in stores or bars since the laws prohibiting the sale of tobacco products to minors are strictly enforced. It is pretty difficult to purchase cigarettes unless you can find a “pusher” [peddler] and buy from him. (FGD 1)*

However, some participants highlighted that there is poor enforcement of the sale of cigarettes in community bars in rural areas. They also mentioned that children sometimes use the excuse that their parents or uncles have sent them as a decoy to purchase cigarettes for themselves. A child in one of the FGDs in a rural area made the following statements:

*In small towns like ours, cigarette vendors do not question children when they enter their stores or bars to make purchases, and even if they do, children explain that they are purchasing cigarettes for their parents, uncles, or other family members. This makes it easy for children to buy cigarettes. Since several children have smoking relatives, it can occasionally be challenging to tell if they are telling the truth. (FGD 2)*

### Smoking of marijuana

FGDs with children revealed that children smoke marijuana and often hide when smoking. Consequently, they indicated that it is difficult to estimate the proportion of children engaged in marijuana smoking. The quote below corroborates the theme:

*A few children I know smoke “wee” [name of marijuana in the local language]. Children who smoke ‘wee’ often transitioned from smoking cigarettes for years to “wee” smoking. Since it is illegal to even for adults to smoke ‘wee,’ it is pretty challenging to know the magnitude of children engaged in ‘wee’ smoking in this area. However, it is common knowledge that some children smoke it. (FGD 3)*

### Smoking of waterpipe/shisha

A key informant revealed that girls are increasingly smoking waterpipe/shisha. He defined waterpipe/shisha as a smoking technique using one or more stemmed instruments to smoke flavour or unflavoured tobacco. He added that before the stemmed smoke reaches the smoker, it passes through water or other liquids. He elaborated that the primary cause of the increased patronage of waterpipe/shisha is the myth that the flavoured or unfavoured tobacco used in waterpipe/shisha and the water that the stemmed smoke passes through makes waterpipe/shisha smoking less risky than cigarette smoking.

*Waterpipe/shisha was unknown in our part of the world until recently. But it has recently become popular among young people, including children, in the urban areas. Although smoking is uncommon among Ghanaian women, waterpipe/shisha use is more widespread among young urban females. This is due to the erroneous belief that shisha is less harmful than cigarettes and other illicit substances. (KII 1)*

### Predictors of smoking among children

Regarding educational attainment, not in school and primary school were merged since not in school had few cases. Table 4 presents the predictors of smoking among children. Children aged 8–10 (AOR = 0.178; 95% CI = 0.050–0.633; p-value = 0.008), children aged 11–13 (AOR = 0.253; 95% CI = 0.105–0.614; p-value = 0.002), children residing in the Middle belt (AOR = 0.361; 95% CI = 0.248–0.526; p-value = < 0.001), and children residing in the Northern zone (AOR = 0.153; 95% CI = 0.081–0.291; p-value = < 0.001) were less likely to have ever smoked.

**Table 4:** Predictors of smoking among children

Variables	AOR	95% CI	P-values
*Sex*			
Male	8.819	5.373–14.475	< 0.001
Female (RC)			
*Age*			
8–10	0.178	0.050–0.633	0.008
11–13	0.253	0.105–0.614	0.002
14–17 (RC)			
*Educational attainment*			
No education/Primary (RC)			
JHS	0.721	0.255–2.034	0.536
SHS	1.935	0.628–5.963	0.250
Tertiary	2.711	0.797–9.224	0.111
*Religion*			
Christianity (RC)			
Islam	0.750	0.352-1.600	0.457
Other	1.160	0.501–2.685	0.729
*Ecological zone*			
Coastal zone (RC)			
Middle belt	0.361	0.248–0.526	< 0.001
Northern zone	0.153	0.081–0.291	< 0.001
*Living arrangements*			
Both biological parents (RC)			
Biological mother alone	0.876	0.552–1.391	0.574
Biological father alone	1.850	1.022–3.347	0.042
Other	1.124	0.731–1.727	0.595
*Currently working*			
Yes	1.531	0.918–2.554	0.103
No (RC)			

AOR = Adjusted Odds Ratio; RC = Reference category

Also, male children (AOR = 8.819; 95% CI = 5.373–14.475; p-value = < 0.001) and children living with their biological fathers (AOR = 1.850; 95% CI = 1.022–3.347; p-value = 0.042) were more likely to have ever smoked.

### Reasons for and factors that lead children to smoke

Children aged 8–17 were asked to give the reasons why children engage in smoking. From Table 5, a little over two-fifth (41.6%) of participants reported they smoked for fun, while two fifth (40.4%) of participants smoked due to group culture. About one-tenth (9.9%) of participants smoked to get ‘high,‘ while about 6% smoked to relieve boredom or anxiety. Furthermore, about 2% of participants smoked out of curiosity.

**Table 5:** Information regarding reasons and factors initiating children into smoking

Responses	Male (%)	Female (%)	Total (%)
*Reasons for smoking*			
Get high	13 (9.2)	3 (15.8)	16 (9.9)
For fun	57 (40.1)	10 (52.6)	67 (41.6)
Group culture	60 (42.3)	5 (26.3)	65 (40.4)
Kill boredom/anxiety	9 (6.3)	1 (5.3)	10 (6.2)
Curiosity	3 (2.1)	0 (0.0)	3 (1.9)
*Factors that lead children to smoke*			
Introduced by friends	80 (57.6)	10 (55.6)	90 (57.3)
Family member smokes	28 (20.1)	6 (33.3)	34 (21.7)
Out of curiosity	18 (12.9)	2 (11.1)	20 (12.7)
Adverts and videos (movies, dramas, etc.).	13 (9.4)	0 (0.0)	13 (8.3)

The study also examined the factors that lead children to smoke. Table 5 shows that over half (57.3%) of participants reported that their friends influenced them to smoke. In comparison, a little over one-fifth (21.7%) of participants were influenced by family members who smoke to initiate smoking. A little over one-fifth (12.7%) of participants were influenced by curiosity to smoke, while less than one-fifth (8.3%) were motivated by advertisements and videos on smoking to initiate smoking.

During the focus group discussions, children aged 8–17 were also asked to provide their thoughts on what they considered were the main drivers influencing children to initiate smoking cigarettes and other tobacco products. The children mentioned that the main drivers of smoking among children were having friends, parents, other family members, or neighbours who smoked. For most participants having friends who smoke has a compelling influence in making them cultivate the habit of smoking. This finding supports the quantitative data that having a friend who smokes influences children to initiate smoking. Below are the views of participants:

*Most children belong to groups. They join groups at school, and they also have friends in their neighbourhoods. If a child or a member of the group smoke, they may persuade other members to try smoking. Once children start smoking, they may become addicted to it. (FGD 4)*

*Some children smoke to be accepted within their group of friends because of fear that their smoking friends won’t accept them [non-smoking members]. So for many children, smoking has become a means to be accepted and retain their group memberships. (FGD 5)*

The FGDs also highlighted that having parents or other family members who smoke has a compelling influence in making a child stick to smoking as a habit. This finding is similar to quantitative findings that family members who smoke influence children to initiate smoking. One child explained that:

*All the men in our home, including my father, uncle, and brothers, smoke. They usually send me to go out to buy their cigarettes. I occasionally take a couple when they send me or use my money to try it out for fun and to have a feel of it. (FGD 6)*

Another child, whose uncle had introduced him to smoking, narrated the following:

*I’m close with my younger uncle, and we get along well. He smokes a lot if I go with him somewhere. On one occasion, he offered me a cigarette in exchange for my pledge not to tell anyone. (FGD 7)*

### Where children smoke

According to the qualitative findings, children in Ghana do not openly smoke in public spaces. Participants in the FGDs said that many children who smoke pick covert locations since they know smoking is unacceptable. The FGDs show that areas such as deteriorating, unfinished, and abandoned buildings make up sites where children typically hide out to smoke. Others reported that some children smoke in the bushes, on beaches, dark places at night, and in bars. The following quotes by some participants in the FGDs corroborate the theme:

*Smoking is common among children in this town, but they hide to do it at the beach. If you go there right now, you will see it for yourself. Some children from the surrounding SHS schools remove their uniforms and pretend they are ordinary children from the Edina community. (FGD 8)*

*Numerous reports of children smoking marijuana in the past few years have been made to the school administration. The school is quite concerned about this. Our Senior Housemaster, who is a disciplinarian, smart, and aware of some of the places where students hide to smoke, occasionally walks around the school’s campus, checking for anyone who might be smoking. His actions have led to a decrease in its occurrence, although some students continue to smoke in places where it is more difficult to monitor them. (FGD 9)*

In the Komenda Edina Eguafo Abirem (KEEA) Municipality, a key informant reported that children who smoke marijuana are occasionally arrested during police swoops. He explained that:

*The police occasionally make swoops to arrest persons who smoke ‘wee’ in various smoking hiding places. By chance, some of these individuals are very young, including children. They hide in bushes, abandoned buildings, and sometimes kiosks to smoke. There is a church building here, and it is in a secluded place. When church service is over, that is when young people, including children, go to smoke. The sad thing is that it includes a few girls. It’s really a bothersome development. (KII 2)*

### Regulation of smoking among children

KIIs with the staff of government agencies revealed that smoking among children is fast becoming a challenge despite regulations prohibiting the sale of cigarettes to persons under 18. They reported that the government is concerned, and through its agencies, it is taking measures to address it. A staff of DOC noted that smoking among children constitutes a vital subject/theme in their public outreach campaigns. The key informant narrated that:

*Tobacco use among children also constitutes a grave concern for the country. We have received reports and believe some children in the Senior High School smoke marijuana, cigarettes, and shisha. It has also been reported that about 90% of cases at the Accra Psychiatric Hospital annually are drug abuse related, and most cases are young people, including children. These reports provide enough evidence to suggest that substance abuse is gaining ground in Ghana among the youth, including children, so we need to act quickly to salvage the situation. The NCCE, DOC, DSW, and their CSO partners may have to intensify their educational drive on substance abuse, which seems to be a rising trend among young people in the country. (KII 3)*

Another key informant highlighted the need for the government to adequately resource them to help perform their responsibilities, which will help curb smoking among children.

*Since smoking is not tolerated in Ghana, not even among adults, reports of children smoking are alarming. As a child related agency, we work closely with our law enforcement partners to encourage reporting and investigating any concerns about the possession and use of illegal substances so that the necessary action can be taken to stop youth smoking before it starts. We know it will be challenging, but we must nonetheless complete it. However, we shall implore the government to provide the necessary material and financial backing to fulfil our responsibilities properly. (KII 4)*

Another key informant explained that shisha is prohibited in Ghana despite its usage among the general population, including children.

*By law, the Food and Drugs Authority (FDA) must regulate the use of shisha. This requires operators to come to register before offering it to the public. To date, no one has come to us to register. This means anyone providing shisha to the public is engaged in illegality (KII 4)*.

A key informant at the Narcotics Control Board (NACOB) indicated that the Narcotic Drugs (Control, Enforcement, and Sanctions) Law 1990 (PNDCL 236) bans narcotic drugs and establishes the NACOB to stem the flow of drugs into the country. The key informant also mentioned that Ghana has also signed and ratified many United Nations Conventions and Protocols on drugs, such as the 1961 Single Convention, 1971 Convention on Psychotropic Substances, 1972 Protocol Amending the 1961 Single Convention, and 1988 Convention against Illicit Trafficking of Narcotic Drugs and Psychotropic Substances. In line with UN guidelines, all signatories, including Ghana, must follow guidelines set out to tackle issues related to drug control and controlled substances that affect younger people, especially children. In effect, the use of psychotropic drugs is an area the government strives to fight not only among children but also among adults. An official at the Commission on Human Rights and Administrative Justice summed up the crucial steps taken by the government to confirm the narrative:

*The Ghanaian government has made significant efforts to combat smoking among young people, including children. Legal measures, such as the passage of national laws and the ratification of international treaties, are some of the steps adopted to regulate the use of tobacco products among children in the country. The government has also implemented institutional strengthening, increased awareness-raising efforts across all regions, and developed child protection policies. (KII 5)*

## Discussion

The study aimed to examine the prevalence of smoking, the kinds of substances children smoke, predictors of smoking, reasons for and factors that lead children to smoke, and regulation of smoking among children in Ghana. The findings indicate that 3.2% of children had ever smoked, and it is more prevalent among male children (5.5%) than female children (0.8%). On the one hand, the prevalence of smoking among children in this study was low compared to earlier studies in Serbia [15] and Malaysia [14]. For instance, Veres et al.‘s [15] in Serbia among high grade primary students found that 35% of students had ever smoked. Norbanee et al.‘ [14] study in Malaysia among primary school children found that 25% of children had ever smoked. On the other hand, the prevalence of smoking among children in this study was high compared to a study in Brazil [16]. Silva et al.‘s [16] study among children aged 7–17 in Brazil found that 2.4% of children had ever smoked. The differences in smoking prevalence among children could be attributed to differences in the sample population, respondents’ ages, and measurements. For instance, Silva et al.‘s [16], Norbanee et al.‘ [14], and Veres et al.‘s [15] studies were conducted among school going children, while this study was conducted among both school going and non-school-going children. Also, Veres et al.‘s [15] study was conducted among children aged 11–15, while Silva et al.‘s [16] and Norbanee et al.‘ [14] studies were conducted among children aged 7–17.

The study also found that male children reported higher smoking prevalence than female children. The multivariate analysis also showed that male children are more likely to have ever smoked. These findings are similar to those of earlier studies [1, 17, 18]. A plausible explanation is that males are more likely to engage in risky behaviour than females [19, 20].

Furthermore, smoking was more prevalent among older children than younger children. The multivariate analysis also showed that children aged 8–10 and 11–13 were less likely to have ever smoked. This finding supports earlier studies that smoking is more prevalent in older children than younger children [14, 16]. Older children may be more exposed to tobacco products and are, therefore, more likely to try smoking than younger children.

Children residing in the Coastal zone were more likely to have ever smoked than children living in the Middle belt and Northern zone. Greater Accra region is situated in the Coastal zone, and studies have found that the prevalence of smoking is highest in the region [1, 11]. Therefore, the high prevalence of smoking in the Greater Accra region may explain why children in the Coastal zone are more likely to have ever smoked than children residing in other ecological zones.

According to the study, cigarette smoking (78.3%) is far more common among children in the country than marijuana or shisha usage (18% and 3.7%, respectively). This supports earlier findings by St Claire et al. [21], which found that cigarette smoking is the most popular smoking habit among children. The results also highlighted sex differentials in smoking, with cigarettes and marijuana smoking being more prevalent among male children, while shisha smoking was more prevalent among female children. Shisha smoking was more prevalent among female children because of the perception that it is less harmful compared to cigarettes and marijuana [22]. Females are often less likely to take risks than males [19, 20].

The study also revealed that some children who used marijuana had a history of smoking cigarettes, supporting previous research that indicates an association between marijuana and cigarette use [23]. This finding suggests a connection between smoking cigarettes and switching to marijuana use.

Regarding the frequency of smoking, the majority of children (57.1%) had smoked once, 32.3% had smoked repeatedly, 5.6% were unable to recall how many times they had smoked, and 5% had smoked twice. Despite the low prevalence of smoking among children, it is worrisome that 32.3% had smoked repeatedly, and 5.6% could not recall how frequently they had smoked. Another concerning finding is that over 2 out of 10 children who had ever smoked also smoked marijuana. These findings urgently call for policy makers to strategise and strengthen their policies, programmes, and interventions to address smoking among children.

A little over four out of ten children smoked for fun and due to group culture. Children also occasionally smoked to get high, to kill boredom or to calm their nerves, or just out of curiosity. Also, children were influenced by friends; parents, family members, and neighbours who smoke; curiosity; and advertisements and videos to initiate smoking. These findings corroborate previous studies, which found that friends [14, 15, 17, 24], parents, family members, and neighbours who smoke [14, 15], curiosity [24, 25], and advertisements [3] influenced people to initiate smoking. These findings highlight the need for parents, relatives, and neighbours who smoke to be mindful of their smoking habits since it negatively impacts children’s smoking habits. Also, parents and guardians need to show interest in who their children spend time with or associate with. Policy makers should enforce the Tobacco Control Regulations 2016 (L.I. 2247), which regulates the advertisement, promotion, and sponsorship of tobacco products.

The multivariate analysis also found that children who live with their biological fathers are more vulnerable to smoking than those living with both biological parents. The lack of proper supervision by fathers may explain this phenomenon.

The qualitative findings further show that some children started smoking because they used to run cigarette deliveries for their family members, which offered them a chance to try it out. This situation strengthens the case for enforcing the Tobacco Control Regulations 2016 (L. I. 2247) and the Public Health Act 2012 (Act 851), which prohibits the sale of cigarettes to persons under 18. This finding supports an earlier study, which found that access to substance smoking products, such as e-cigarettes, influence smoking [26].

The qualitative findings point to various locations where children prefer to smoke, and many are hidden from the general public. Children’s smoking areas include deteriorating, incomplete, and abandoned structures, as well as bushes, beaches, dark places at night, bars and places far from their residences. Most children smoke in secret locations since they know smoking is considered a deviant act in Ghana. This finding supports Glenstrup et al.‘s [27] study, which found that adolescents who smoke feel discomfort when their smoking behaviour is exposed, so they keep their smoking habits hidden from the public.

The government has made some progress in passing laws, ratifying international treaties, establishing entities, raising public awareness, and putting in place controls over the use and distribution of tobacco products, which is typically what most children in the country smoke. Smoking among children is still prevalent despite the preventive measures taken and the government prohibiting the sale and use of tobacco products among children. According to the findings, this is typically caused by the lack of funding for government organisations to monitor and regulate measures that deter children from smoking. Although Ghanaian law forbids children from purchasing tobacco products, inadequate regulatory procedures allow children to access marijuana and tobacco products through unlawful channels. Although there is some compliance among specific retailers in urban areas, many tobacco product vendors in rural areas continue to sell to children out of ignorance or a lack of regard for law and order [28, 29].

### Strengths and limitations of the study

The study had some strengths. Key among them is the large sample size, which was nationally representative. The study also had a high response rate among children in all ten regions. This study significantly advances the knowledge of smoking among children and the factors that contribute to it. The results might be helpful for programming, regulation, and strategy for the country and other sub-Saharan countries. Despite the strengths, the study also has some limitations. First, children were asked if they had ever smoked. Since smoking is deemed a deviant act and prohibited by Ghana’s law among persons below 18 years, children may under-report their smoking habits. Second, there may be recall bias since children were to recollect their smoking experience, which may result in underreporting of their smoking encounters. Third, this study is cross-sectional; therefore, causal relationships between the dependent and independent variables cannot be established. Fourth, the secondary data used for this study is 5 years old; therefore, the substance smoking behaviour of children in Ghana may have changed. Despite the data being 5 years old, this study is warranted since it is essential to understand the past magnitude of substance abuse among children to serve as a reference point for policymakers to measure the success of their interventions implemented over the years to curb substance smoking among children. Fifth, the study did not ask children about their use of e-cigarettes, heat-not-burn and smokeless tobacco products.

## Conclusions

The study indicated that the prevalence of smoking was low among children aged 8–17. Despite the relatively low prevalence of smoking among children in Ghana, this issue remains a cause for concern due to its illegality and the severe implications it poses on the health and wellbeing of children. The study also found that children who have ever engaged in smoking reported using cigarettes, marijuana, and shisha. Children were influenced by parents, peers and neighbours to engage in smoking. This study points to the need for an enhanced educational effort to improve children’s awareness of the risks associated with adolescent smoking in school and other settings. To prevent future smoking addiction in children, educating them about the dangers of smoking while they are still young is essential. The National Commission for Civic Education, Ghana Education Service, Ministry of Gender, Children and Social Protection, and their allies must support this educational initiative.

### Electronic supplementary material

Below is the link to the electronic supplementary material.

Supplementary Material 1

## Data Availability

The datasets generated and/or analysed during the current study are not publicly available due to them containing information that could compromise research participant privacy but are available from the corresponding author on reasonable request.

## List of abbreviations

CSO: Civil Society Organisation
DOC: Department of Children
DSW: Department of Social Work
EAs: Enumeration Areas
FDA: Food and Drugs Authority
FGDs: Focus Group Discussions
JHS: Junior High School
L. I.: Legislative Instrument
MICS: Multiple Indicator Cluster Survey
MoGCSP: Ministry of Gender, Children and Social Protection
NACOB: Narcotics Control Board
NCCE: National Commission for Civil Education
NCPC: National Child Protection Committee
RC: Reference Category
SHS: Senior High School
SPSS: Statistical Package for Social Sciences
