# Supplementary material for: Assessment of prevalence, predictors, reasons and regulations of substance smoking among children in Ghana
Source: BMC Public Health. 2023 Nov 16;23:2262. doi: 10.1186/s12889-023-17187-1 (PMC10655291; doi:10.1186/s12889-023-17187-1)
Supplement: Supplementary file 1 — Supplementary Material 1 [file 12889_2023_17187_MOESM1_ESM.docx]

**DEPARTMENT OF CHILDREN – MINISTRY OF GENDER CHILDREN AND SOCIAL PROTECTION**

**SITUATIONAL ANALYSIS OF CHILDREN IN GHANA 2018**

***CHILD QUESTIONNAIRE***

**A. GENERAL INFORMATION**

| IDENTIFICATION |
| --- |
| 1. Name..............................................................................................................................................…………  2. Sex 1. Male 2. Female  3. Date of birth 🞎🞎🞎🞎🞎🞎  D D / M M / Y Y 99. Don’t Know  4. Age **[IN COMPLETED YEARS]**..................... ........ 99. Don’t Know  5. Place of Residence ………………………………………………………………………………………………….. |

6. Do you have a birth certificate? **[NOT IMMUNISATION CARDS]**

1. Yes 2. No 99. Don’t Know

7. Who do you live with and is responsible for you?

1. Both biological Parents **[GO TO Q9]** 7. Elder Sibling

2. Biological Mother alone 8. Grandparent/s

3. Biological Father alone 9. Guardian not related to respondent

4. Relative of mother 10. Apprentice master

5. Relative of father 11. Other **[SPECIFY]**...........................................

6. Independent

8. What are your reasons for not living with **both** your biological parents?

1. Lack of financial support from parents 7. Parents overseas

2. Easy access to school 8. Ran away from home

3. Death of supporting parent 9. Death of both parents

4. Abandoned by parents 10. Apprenticeship away from home

5. Parents working elsewhere 11. Other **[SPECIFY]**.................................

6. Parents divorced/separated 99. Don’t Know

9. What is the main occupation of your father? ....................................................................................................

99 Don’t Know

10. What is the main occupation of your mother?....................................................................................................

99 Don’t Know

11. **CHECK Q7:**

**🞏** **LIVES WITH BOTH PARENTS [GO TO Q12]**

**🞏NOT LIVING WITH BOTH PARENTS**

**↓**

If not living with both parents what is the main occupation of your guardian?..................................................

99 Don’t Know

12. What is your religion?

1. Christianity 4. No Religion

2. Islam 5. Other **[SPECIFY]**.............................……….

3. African Traditionalist 99. Don’t Know

13. What is the main religion of your family?

1. Christianity 4. No Religion

2. Islam 5. Other **[SPECIFY]**.............................…………

3. African Traditionalist 6. Mixed **[SPECIFY]**………………………………

99. Don’t Know

14. Which ethnic group do you belong to?

1. Ashanti 5. Ga 9. Mole

2. Akwapim 6. Adangbe 10. Dagbani

3. Fanti 7. Ewe 11. Nzema

4. Ahanta 8. Guan 12. Other **[SPECIFY]** ………………………………

99. Don’t Know

15. Where were you born?

1. In this town/village 4. Greater Accra 9. Eastern 99. Don’t Know

2. In another town/village in 5. Ashanti 10. Volta

this District 6. Brong Ahafo 11. Northern

3. In another District in this 7. Western 12. Upper West

Region 8. Central 13. Upper East

16. Where is your current place of residence?

1. In this town/village 4. Greater Accra 9. Eastern

2. In another town/village in 5. Ashanti 10. Volta

this District 6. Brong Ahafo 11. Northern

3. In another District in this 7. Western 12. Upper West

Region 8. Central 13. Upper East

17. Have you always lived there?

1. Yes **[GO TO SECTION B. FAMILY/HOUSEHOLD INFORMATION ON PAGE 3]**

2. No

99. Don’t Know **[GO TO SECTION B. FAMILY/HOUSEHOLD INFORMATION ON PAGE 3]**

18. How long have you lived there? .......................................................................................

99. Don’t Know

19. Where did you move from?

1. Another town/village in 3. Greater Accra 8. Eastern 99. Don’t Know

this District 4. Ashanti 9. Volta

2. Another District in this 5. Brong Ahafo 10. Northern

Region 6. Western 11. Upper West

7. Central 12. Upper East

20. **CHECK Q16:**

**🞏** **LIVING IN THE SAME REGION [GO TO SECTION B. FAMILY/HOUSEHOLD INFORMATION ON PAGE 3]**

**🞏 LIVING IN A DIFFERENT REGION/MOVED FROM A DIFFERENT REGION**

**↓**

Why did you move from where you were?

1. Parents are unable to care for me 5. In order to take care of parents

2. To earn own income 6. Parental neglect

3. Single parent could not take 7. Parents transferred or moved from previous abode

care of me with income 8. Other **[SPECIFY]**................................................

4. To get money to further education 99. Don’t Know

**B. FAMILY/HOUSEHOLD INFORMATION**

1. How many relatives do you live with in your household? ………………….………………………………………..

|  | 1. Boys | 2. Girls | 99. Don’t Know |
| --- | --- | --- | --- |
| 2. How many of them are children? |  |  |  |
| 3. How many of these children are older than you? |  |  |  |
| 4. How many of these children are younger than you? |  |  |  |
| 5. How many of these children are your siblings? |  |  |  |

|  | 1. Boys | 2. Girls | 3. None |
| --- | --- | --- | --- |
| 6. How many househelps living in your household are children? |  |  |  |

7. Who is your care-giver?

1. Mother **[GO TO Q10]** 7. Househelp

2. Father **[GO TO Q10]** 8. Brother/male cousin

3. Grandmother 9. Sister/female cousin

4. Grandfather 10. Unrelated elder person

5. Aunt 11. No one **[GO TO Q10]**

6. Uncle 12. Other **[SPECIFY]**................................................

8. What is the age of your care-giver? ……………………………………………………………..

99. Don’t Know

9. What is the educational background of your care-giver?

1. Never been to school 5. Vocational/Technical

2. Primary 6. Tertiary

3. Middle School/JSS 7. Non-Formal Education

4. Secondary/SSS 99. Don’t Know

10. What is the main corrective method used at home? **[MULTIPLE RESPONSES]**

A. Caning/Whipping F. Extra household chores

B. Denial of food/pocket money G. Counselling/Advice

C. Scolding H. None

D. Physical punishment with bare I. Confinement/restriction of freedom

hands (slapping, hitting, beating) J. Other **[SPECIFY]** .........................................................

E. Public shaming

11. Who usually carries out the correction in the home? **[MULTIPLE RESPONSES]**

A. Father E. Unrelated elder person

B. Mother F. Any related elder person

C. Elder Sibling G. Other **[SPECIFY]**...............................................

D. Care-giver (not parent or elder sibling)

| 12. What type of chores do you perform?  **[MULTIPLE RESPONSES]** | Time of Day of Chore Performance  **[MULTIPLE RESPONSES]**  **M(orning) (5am – Noon)**  **A(fternoon) (Noon – 6pm)**  **E(vening) (6pm – 10 pm)**  **N(ight) (10pm – Midnight)**  **P(redawn) (Midnight – 5am)** | Frequency  **x/D(ay)**  **x/W(eek)**  **x/M(onth)** |
| --- | --- | --- |
| A. Fetching of water  B. Fetching of firewood  C. Cooking and washing of dishes  D. Assisting parents on the farm  E. Sweeping  F. Washing of clothes  G. Selling of ice-water  H. Other **[SPECIFY]**....................................... | ..........................................................................................................................................................................................................................................................................................…………………………………..............................………….. | ........................................................................................................................................................………………......... |

13. How does the performance of chore(s) affect you? **[MULTIPLE RESPONSES]**

A. School performance suffers F. Physical problems like backache

B. Insufficient time to study or do homework G. No deleterious effect

C. School attendance suffers H. Learn to cook

D. No time for leisure, games or friends I. Learn to sew

E. Always feel tired J. Other **[SPECIFY]**...............................................

14. Do you care for or look after someone younger than yourself?

1. Yes

2. No **[GO TO Q16]**

15. If Yes, who do you look after? **[INDICATE IN TABLE BELOW]**

| Relationship to person cared for | Age | What is the reason for your looking after this person? |
| --- | --- | --- |
| 1. .........................................................  2. ......................................................... | ............................................ | .............................................................................................  ............................................................................................. |

16. Do you enjoy living in your household? 1. Yes 2. No 99. Don’t Know

17. Give reasons for your answer in Question 16 ...........................................................................................................

..................................................................................................................................................................................

**C. LIVING CONDITIONS**

1. Where do you normally sleep?

1. In a house (bedroom) 5. In house (roof top) 8. Lorry station/Bus stop

2. In a house (living room) 6. Pavement 9. Uncompleted house/structure

3. In a house (kitchen) 7. Kiosk/Store 10. Other **[SPECIFY]** ...................................

4. In a house (verandah)

2. Do you normally sleep there alone? 1. Yes **[GO TO Q5]** 2. No

3. If No, who do you normally sleep with? **[MULTIPLE RESPONSES]**

A. Mother G. Sister

B. Father H. Brother

C. Grandmother I. Other Relatives

D. Grandfather J. Househelp

E. Aunt K. Unrelated Person

F. Uncle

4. How many people sleep in the place where you sleep?......................................................................................

5. What do you normally sleep on?

1. Bed 4. Plastic sheet 7. Bare floor

2. Mattress 5. Sack 8. Cardboard

3. Straw mat 6. Other **[SPECIFY]** ..............................................

6. What is the main construction material your house is made of? **[EXTERIOR STRUCTURE]**

**[IF IN RESPONDENT’S DWELLING PLACE AND RESPONDENT DOES NOT KNOW, OBSERVE AND INDICATE THE MATERIAL ACCORDINGLY]**

1. Mud/Clay 4. Aluminium Sheets 7. Brick

2. Sancrete 5. Bamboo/Rafia 99. Don’t Know

3. Wood 6. Other **[SPECIFY]**..............................................

7. What is the main lighting system used in your house?

1. Electric lamp 4. Torch light 7. Other **[SPECIFY]**......................

2. Candle 5. Kerosene lamp

3. Gas lamp 6. None

8. Is the lighting sufficient for your own use?

1. Yes **[GO TO Q10]** 2. No 99. Don’t Know

9. If No or Don’t Know, give reasons ............................................................................................................................

10. Where do you rest or play around the house? **[MULTIPLE RESPONSES]**

A. Courtyard D. Kitchen F. No space at home

B. Bedroom E. Garden G. On the street outside the house

C. Living Room H. Other **[SPECIFY]** .................................

11. What is your main source of drinking water?

1. Pipe-borne water 3. Bore hole/well 5. Water Tank

2. Stream/Spring/Pond 4. Rainwater 6. Other **[SPECIFY]** ......................

12. How long does it normally take you to get there, get drinking water and come back home?

1. Less than 5 minutes 4. 30 to 45 minutes 99. Don’t Know

2. 5 to 15 minutes 5. 45 minutes to 1 hour

3. 15 to 30 minutes 6. Over 1 hour

13. How much do you pay per bucket of water?

1. ¢20 3. ¢100 5. ¢200 7. Nothing

2 ¢50 4. ¢150 6. Over ¢200 8. Other **[SPECIFY]** ………...

99. Don’t Know

14. What is your main source of laundry/washing water?

1. Pipe-borne water 3. Bore hole/well 5. Water Tank

2. Stream/Spring/Pond 4. Rainwater 6. Other **[SPECIFY]** ......................

15. How long does it normally take you to get there, get the laundry/washing water and come back home?

1. Less than 5 minutes 3. 15 to 30 minutes 5. 45 minutes to 1 hour

2. 5 to 15 minutes 4. 30 to 45 minutes 6. Over 1 hour

99. Don’t Know

16. What is your main source of bathing water?

1. Pipe-borne water 3. Bore hole/well 5. Water Tank

2. Stream/Spring/Pond 4. Rainwater 6. Other **[SPECIFY]** ......................

17. How long does it normally take you to get there, get the bathing water and come back home?

1. Less than 5 minutes 3. 15 to 30 minutes 5. 45 minutes to 1 hour

2. 5 to 15 minutes 4. 30 to 45 minutes 6. Over 1 hour

99. Don’t Know

18. Who normally fetches water? **[MULTIPLE RESPONSES]**

A. Boys C. Adults E. Other **[SPECIFY]** ..................................

B. Girls D. Not Applicable

19. What is the method of disposal of your household rubbish?

1. Burning 5. Indiscriminate disposal **[GO TO Q22]**

2. Dumping (Crude Rubbish Dump) 6. Refuse Collection Service

3. Dumping (Official Rubbish Dump) 7. Other **[SPECIFY]** …………………………………

4. Burying

20. Where is your household rubbish disposed of? ....................................................................................................

21. How long does it normally take you to get to the place, dump the rubbish and come back home?

1. Less than 5 minutes 3. 15 to 30 minutes 5. 45 minutes to 1 hour

2. 5 to 15 minutes 4. 30 to 45 minutes 6. Over 1 hour

99. Don’t Know

22. Who disposes the rubbish in the household? **[MULTIPLE RESPONSES]**

A. Boys C. Adults

B. Girls D. Other **[SPECIFY]** ...........................................

23. What type of toilet facility do you have at home?

1. KVIP 3. Pit Latrine 5. Pan Latrine

2. Water Closet 4. No facility **[GO TO Q27]** 6. Other **[SPECIFY]** .........................

24. Is this home toilet facility used only by your own household or is it shared with other households?

1. Used only by own household 2. Shared with other households

25. Do you use this home toilet facility?

1. Yes **[GO TO Q30]** 2. No

26. Why do you not use the toilet facility in your home?

.................................................................................................................................................................................

……..........................................................................................................................................................................

27. Do you use the public toilet facility?

1. Yes 2. No **[GO TO Q31]**

28. Do you pay for the use of the public toilet?

1. Yes 2. No **[GO TO Q30]**

29. How much do you pay per visit?

1. ¢20 3. ¢100 5. ¢200 7. Other **[SPECIFY]** ……..

2. ¢50 4. ¢150 6. Over ¢200 99. Don’t Know

30. How long does it normally take you to get to the toilet facility?

1. Less than 5 minutes 3. 15 to 30 minutes

2. 5 to 15 minutes 99. Don’t Know

31. Where do you bathe?

1. Bathroom **[GO TO Q34]** 3. Paying bath house

2. Nearby stream/river **[GO TO Q33]** 4. Other **[SPECIFY]** .........................................................

**[GO TO Q33]**

32. How much do you pay for the use of the paying bath house per visit?

1. ¢20 3. Over ¢50

2. ¢50 4. Other **[SPECIFY]** ........................................................................

33. How long does it normally take you to get to the bathing place?

1. Less than 5 minutes 3. 15 to 30 minutes

2. 5 to 15 minutes 99. Don’t Know

34. Where is the household cooking done?

1. Kitchen 4. Courtyard 6. Verandah

2. Living Room 5. Corridor 7. Don’t cook **[GO TO Q38]**

3. Bedroom 8. Other **[SPECIFY]** ..............

35. What is the main source of fuel for cooking? **[IF SOURCE IS NOT FIREWOOD, GO TO Q38]**

1. Electricity 3. Kerosene 5. Firewood

2. Gas 4. Charcoal 6. Other **[SPECIFY]** ..................................

36. How long does it normally take you to get there, gather firewood and come back home?

1. Less than 5 minutes 4. 30 to 45 minutes 7. Purchased from seller

2. 5 to 15 minutes 5. 45 minutes to 1 hour 99. Don’t Know

3. 15 to 30 minutes 6. Over 1 hour

37. Who collects the firewood? **[MULTIPLE RESPONSES]**

A. Boys C. Adults

B. Girls D. Other **[SPECIFY]** ..........................................

38. On the whole, are you happy with your household amenities? **[NOTE: THIS QUESTION REFERS TO ALL AMENITIES, AND NOT JUST FUEL]**

1. Yes 3. Some of the time

2. No 99. Don’t Know

39. Give reason(s) for your answer in question 38........................................................................................................

..................................................................................................................................................................................

**D. EDUCATION**

1. Are you currently attending any school?

1. Yes **[GO TO Q8]**

2. No

**NON SCHOOL-GOERS**

2. Why are you not attending school?

1. Completed 5. Dropped out

2. Suspended by school 6. Never been to school **[GO TO Q4]**

3. Suspended by self 7. Apprenticeship**[GO TO SECTION E. APPRENTICESHIP ON PAGE 12]**

4. Other **[SPECIFY]**.............................................

3. What is your highest educational level/stage attained? **[INDICATE SPECIFIC CLASS]** ..................................

99. Don’t Know

4. **CHECK SECTION A. GENERAL INFORMATION, Q4:**

**🞏** **16 YEARS AND ABOVE [GO TO Q6]**

**🞏 15 YEARS AND BELOW**

**↓**

What are your reasons for not attending school? **[EXCEPT FOR RESPONSE 1, GO TO Q6]**

1. Lack of interest 5. Got pregnant

2. Lack of parental/family support 6. Got married

3. Lack of school facilities 7. Difficulty in paying fees

4. Withdrawn from school by parents 8. Other **[SPECIFY]**............................................................

5. Why are you not interested in school?

i...........................................................................................................................................................................

ii............................................................................................................................................................................

6. Since you are not attending school, how do you spend your time?

1. Begging 5. Assist parents in their work

2. Roam/wander in town or market 6. Self-employed **[SPECIFY]** ...............................

3. Full-time paid employment 7. Other **[SPECIFY]** .............................................

4. Part-time paid employment

7. If given the opportunity what would you pursue?

1. Learn a trade or enter into apprenticeship

2. Further education

3. Other **[SPECIFY]** .......................................................................................................................................

99. Don’t Know

**[GO TO SECTION F. EMPLOYMENT ON PAGE 14]**

**SCHOOL-GOERS**

8. Do you attend a private or public school?

1. Public school 3. Other **[SPECIFY]**.........................................

2. Private school 99. Don’t Know

9. What class are you at now? …………………………………………………………..

|  | Explain your answer |
| --- | --- |
| 10. Are you interested in schooling?  1. Yes  2. No  99. Don’t Know |  |
| 11. Do you like your school?  1. Yes  2. No  99. Don’t Know |  |
| 12. Do you like your teachers?  1. Yes  2. No  99. Don’t Know |  |
| 13. Do you like the students in your school?  1. Yes  2. No  99. Don’t Know |  |
| 14. Do you like the students in your class?  1. Yes  2. No  99. Don’t Know |  |
| 15. Are there any problems with the school itself?  1. Yes  2. No  99. Don’t Know |  |
| 16. Are there any problems with the teachers?  1. Yes  2. No  99. Don’t Know |  |
| 17. Are there any problems with the students?  1. Yes  2. No  99. Don’t Know |  |

18. How do you usually get to school?

1. Walk 3. Ferry/boat/canoe 5. Other **[SPECIFY]**...................................

2. By vehicle 4. Bicycle

19. How long does it normally take you to get to school from home?

1. Less than 15 minutes 3. 30 minutes to 45 minutes 5. Over 1 hour

2. 15 to 30 minutes 4. 45 minutes to 1 hour 99. Don’t Know

20. How would you describe your academic performance at school?

1. Excellent 3. Average 5. Other **[SPECIFY]**....................................

2. Good 4. Poor

21. How would you describe your performance in sports at school?

1. Excellent 3. Average 5. Other **[SPECIFY]** ....................................

2. Good 4. Poor

22. How would you compare your academic performance with other students in your class?

1. Better 3. Not as good

2. The same 4. Other **[SPECIFY]** ......................................................................................

23 How would you compare your performance in sports with other students in your class?

1. Better 3. Not as good

2. The same 4. Other **[SPECIFY]** ......................................................................................

|  | **[PROBE FOR DETAILS, ELABORATION ON THE CONDITION OF FACILITIES, ADEQUACY OR REASONS FOR RESPONSE]** |
| --- | --- |
| 24. Does your school have a library for your use?  1. Yes  2. No  99. Don’t Know |  |
| 25. Does your school have a workshop for your use?  1. Yes  2. No  99. Don’t Know |  |
| 26. Does your school have water for your use?  1. Yes  2. No  99. Don’t Know |  |
| 27. Does your school have laboratories for your use?  1. Yes  2. No  99. Don’t Know |  |
| 28. Does your school have tables for your use?  1. Yes  2. No  99. Don’t Know |  |
| 29. Does your school have chairs for your use?  1. Yes  2. No  99. Don’t Know |  |
| 30. Does your school have a canteen for your use?  1. Yes  2. No  99. Don’t Know |  |
| 31. Does your school have recreational facilities for your use?  1. Yes  2. No  99. Don’t Know |  |
| 32. Does your school have toilet facilities for your use?  1. Yes  2. No  99. Don’t Know |  |
| 33. Does your school have textbooks for your use?  1. Yes  2. No  99. Don’t Know |  |
| 34. Does your school have teaching aids for your use?  1. Yes  2. No  99. Don’t Know |  |
| 35. Does your school have a stocked first-aid box for your use?  1. Yes  2. No  99. Don’t Know |  |

36. Does your school have sufficient trained teachers?

1. Yes 2. No 99. Don’t Know

37. **CHECK SECTION D. EDUCATION, Q9:**

**🞏** **IN PRIMARY SCHOOL [GO TO Q38]**

**🞏 IN JSS OR SSS**

**↓**

For which subjects are there no trained teachers? **[WRITE ‘NONE’ IF THERE ARE TRAINED TEACHERS FOR ALL SUBJECTS]** ……………………………………………………………………………………………………………………

38. What is the main corrective method used at school? **[MULTIPLE RESPONSES]**

A. Caning D. Counselling G. Other **[SPECIFY]**.................................

B. Suspension/expulsion E. Extra Duties

C. Scolding F. Physical drilling

39. Who normally carries out the method? **[MULTIPLE RESPONSES]**

A. Head teacher C. Prefect

B. Teacher handling incident D. Other **[SPECIFY]**.........................................

40. What corrective method would you consider most appropriate? ...................................................................…….

1. Caning 4. Counselling 7. Other **[SPECIFY]** ……………………….

2. Suspension/expulsion 5. Extra Duties 99. Don’t Know

3. Scolding 6. Physical drilling

41. Why do you consider this the most appropriate method?

..........................................................................................................................................................................…

..............................................................................................................................................................................

42. On the whole, are you happy with your schooling?

1. Yes 3. Some of the time

2. No 99. Don’t Know

43. Give reasons for your answer in question 42 ……………………………………………………………………………

…………………………………………………………………………………………………………………………………

**[GO TO SECTION F. EMPLOYMENT ON PAGE 14]**

**E. APPRENTICESHIP**

1. What craft/trade are you learning? ......................................................................................................................

2. Have you completed basic education?

1. Yes 2. No

3. At what age did you start your apprenticeship?........................................................................................................

4. What is the expected duration of your apprenticeship?.....................................................................................

5. How much was your apprenticeship fee?...........................................................................................................

**[SPECIFY IF PAID IN INSTALMENTS OR AS A LUMP SUM]**

6. Who pays the apprenticeship fee?......................................................................................................................

7. Is there a written apprenticeship agreement with your apprentice master?

1. Yes 2. No 99. Don’t Know

|  | Explain your answer |
| --- | --- |
| 8. Do you like the craft/trade you are learning?  1. Yes  2. No  99. Don’t Know |  |
| 9. Do you like your apprentice master?  1. Yes  2. No  99. Don’t Know |  |
| 10. Do you like your fellow apprentices (if any)?  1. Yes  2. No  99. Don’t Know |  |
| 11. Do you like your place of training?  1. Yes  2. No  99. Don’t Know |  |
| 12. Are you satisfied with apprenticeship?  1. Yes  2. No  99. Don’t Know |  |
| 13. Are there any problems with the place of training?  1. Yes  2. No  99. Don’t Know |  |
| 14. Are there any problems with your apprentice master?  1. Yes  2. No  99. Don’t Know |  |
| 15. Are there any problems with your fellow apprentices (if any)?  1. Yes  2. No  99. Don’t Know |  |

16. How would you describe your performance in your apprenticeship?

1. Excellent 4. Poor

2. Good 5. Other **[SPECIFY]** ........................................................

3. Average

17. How would you compare your performance in your apprenticeship with your fellow apprentices (if any)?

1. Better 3. Not as good

2. The same

|  | **[PROBE FOR DETAILS, ELABORATION ON THE CONDITION OF FACILITIES, ADEQUACY OR REASONS FOR RESPONSE]** |
| --- | --- |
| 18. Does your place of training have water for your use?  1. Yes  2. No  99. Don’t Know |  |
| 19. Does your place of training have toilet facilities for your use?  1. Yes  2. No  99. Don’t Know |  |
| 20. Does your place of training have basic tools/equipment for your use?  1. Yes  2. No  99. Don’t Know |  |

21. What is the main corrective method used at your place of training? **[MULTIPLE RESPONSES]**

A. Caning D. Physical drills

B. Scolding E. Extra duties

C. Counselling F. Other **[SPECIFY]** ......................................................

22. Who normally carries out the method? **[MULTIPLE RESPONSES]**

A. Apprentice master

B. Senior apprentices

C. Other **[SPECIFY]** ...............................................................................................................................

23. What corrective method would you consider most appropriate?

A. Caning D. Physical drills

B. Scolding E. Extra duties

C. Counselling F. Other **[SPECIFY]** ......................................................

24. Why do you consider this the most appropriate method?

………………………………………………………………………………………………………………………………

………………………………………………………………………………………………………………………………

25. Do you receive an allowance?

1. Yes 2. No **[GO TO Q29]**

26. If Yes, who gives you this allowance?

1. Apprentice master 3. Guardian

2. Parent 4. Other **[SPECIFY]** .......................................................................

27. If you get an allowance, do you feel that it is adequate for your needs?

1. Yes 2. No

28. Give reasons for your answer in question 27.......................................................................................................

29. On the whole, are you happy with your apprenticeship?

1. Yes 3. Some of the time

2. No 99. Don’t Know

30. Give reasons for your answer in question 29 ……………………………………………………………………………

…………………………………………………………………………………………………………………………………

**F. EMPLOYMENT**

1. Are you currently doing any paid work?

1. Yes

2. No **[GO TO SECTION G. HEALTH ON PAGE 15]**

2. What kind of paid work do you do?

1. Portering 5. Craft 9. Farming

2. Selling/Hawking 6. Truck Pushing 10. Fishing

3. Househelp 7. Surface mining 11. Bar attendant

4. Trotro mate 8. Sand/stone winning 12. Other **[SPECIFY]** .....................

3. When do you work? **[MULTIPLE RESPONSES]**

A. All day D. Before school everyday G. Harvest/sowing period

B. During vacations E. When school is in shift H. Market days

C. After school everyday F. Fishing season I. On weekends

J. Other **[SPECIFY]** .....................

4. Why do you work? **[MULTIPLE RESPONSES]**

A. Contribute to family income D. To have money to buy needs

B. Bear part of cost of education E. Breadwinner -- to support family

C. To be independent F. Other **[SPECIFY]**.............................................

| 5. How much money do you make? | | | |
| --- | --- | --- | --- |
|  | In a day | In a week | In a month |
| Amount |  |  |  |

6. How would you describe your financial status?

1. Very Good 3. Fair 5. Very Bad

2. Good 4. Bad

7. In the near future, do you expect your financial status to:

1. Improve 3. Decline

2. Remain the same 4. Other **[SPECIFY]** ...................................

8. Do you have any savings? 1. Yes 2. No **[GO TO Q10]**

9. If Yes, where do you save your money?

1. In the bank 4. With a group 7. By myself at home

2. With a SuSu agent 5. With friends 8. Other **[SPECIFY]** ....................

3. With my parents 6. With a guardian

10. How does work affect you? **[MULTIPLE RESPONSES]**

A. Absenteeism from school F. Can afford a better standard of living

B. Feel tired at school G. Greater self-esteem

C. No time to play or relax H. Feel “grown-up”

D. Physical ailments like backache, I. Others respect me more if I have money

muscle aches & pains J. Other **[SPECIFY]** .........................................................

E. Physical injuries (permanent or

chronic conditions)

11. What do you do on week days after work? **[MULTIPLE RESPONSES]**

A. Rest E. Go to the farm I. Go to church

B. Study/Learn F. Go to the market J. Nothing

C. Laundry G. Go to Arabic School K. Other **[SPECIFY]** …………………………

D. Play H. Work

12. What do you do on weekends after work? **[MULTIPLE RESPONSES]**

A. Rest E. Go to the farm I. Go to church

B. Study/Learn F. Go to the market J. Nothing

C. Laundry G. Go to Arabic School K. Other **[SPECIFY]** …………………………

D. Play H. Work

**G. HEALTH**

1. Would you consider yourself physically healthy?

1. Yes 2. No 99. Don’t Know

2. Give reasons for your answer in question 1 ..........................................................................................................

3. **[OBSERVE THE RESPONDENT TO SEE IF THE RESPONDENT HAS ANY PHYSICAL DISABILITY]**

**🞏 NO OBVIOUS PHYSICAL DISABILITY [GO TO Q10]**

**🞏 PHYSICAL DISABILITY OBSERVED OR MENTIONED BY RESPONDENT**

**↓**

1. Hearing impaired 4. Limbs **[SPECIFY]** ............................................................

2. Sight impaired 5. Deformity/Abnormality **[SPECIFY]** .....................................

3. Speech impaired 6. Other **[SPECIFY]** ................................................................

**CHILD WITH PHYSICAL DISABILITY**

4. Do you receive any special assistance or training, having a disability?

1. No **[GO TO Q6]** 2. Yes 99. Don’t Know **[GO TO Q6]**

5. If Yes, what kind of training or assistance do you receive?......................................................................................

6. Do you feel that you are treated equally like the other members of your family?

1. Yes 2. No

7. Explain your answer in question 6 ......................................................................................................................

8. Do you feel that you are treated equally like the other members of your community?

1. Yes 2. No

9. Explain your answer in question 8 .....................................................................................................................

**ALL RESPONDENTS**

10. Where do you usually go to when you are sick (not a minor ailment)?

1. Health Post/Medical Clinic 4. Does not fall sick **[GO TO Q16]** 7. Religious/Sacred Place

2. Herbalist 5. Self-medication 8. Home treatment

3. Drug-store/Pharmacy 6. Hospital 9. Other **[SPECIFY]** .....................

11. Why this form or type of treatment?

1 Proximity advantage 5. Qualified health personnel not available at medical post

2. Has confidence in service 6. My parents take me there

3. Lack of suitable health facilities 7. Other **[SPECIFY]**....................................................

4. Can’t afford cost of formal health service

12. **CHECK Q10:**

**🞏 SELF-MEDICATION [GO TO Q15]**

**🞏** **HOME TREATMENT OR ANY OTHER PLACE**

**↓**

Who takes you there or gives you the home treatment?

1. Alone 3. Elder Sibling 5. Care-giver (unrelated to respondent)

2. Family relation 4. Mother/Father 6. Other **[SPECIFY]** ..................................

13. How long does it normally take you to get to the health-care provider?

1. Less than 15 minutes 3. 30 to 45 minutes 5. Over 1 hour

2. 15 to 30 minutes 4. 45 minutes to 1 hour 99. Don’t know

14. How do you get to the health-care provider?

1. Walk 4. Boat

2. By vehicle 5. Other **[SPECIFY]**..................................................................

3. Bicycle

15. Who pays the health-care provider or for the medication?

1. Parents 4. NGO 7. Relatives

2. Respondent 5. District Assembly 8. Friends

3. Free service 6. Church/Religious Organisation 9. Other **[SPECIFY]**...................... 99. Don’t Know 10. No payment

16. What disease(s) do you suffer most? **[WRITE “NONE” IF NONE, OR “DON’T KNOW” IF RESPONDENT DOES NOT KNOW]**

1...........................................................................................

2...........................................................................................

3...........................................................................................

17. What major disease(s) have you suffered in the last 4 weeks?

1...........................................................................................

2...........................................................................................

3...........................................................................................

18. How does sickness affect you? **[MULTIPLE RESPONSES]**

A. Frequent absence from school D. Feel miserable G. Other **[SPECIFY]** .....................

B. Can’t study E. Can’t work

C. Can’t play F. No effect

19. Have you ever had any sexual experience against your will? 1. Yes 2. No **[GO TO Q27]**

20 If Yes, what form did it take?....................................................................................................................................

21. What age were you then? .....................................................................................................................................

22. Who was that person/s? **[MULTIPLE RESPONSES]**

A. A relative E. Teacher/Training instructor

B. Peer F. Clergy

C. Family friend G. Fetish Priest

D. Stranger H. Other **[SPECIFY]** .......................................................

23. What was the age of the person/s?........................................................................................................................

24. How did you feel after the incident?

1. Enjoyed it 4. Depressed

2. Indifferent 99. Don’t Know

3. Ashamed/hurt

25. What did you do about the incident? ...............................................................................................................

26. How many times have you had sexual experiences against your will?.............................................................

27. Where is the food you normally eat at home prepared? **[MULTIPLE RESPONSES]**

A. Prepared at home D. Bought from a chop-bar

B. Bought on the streets from food stands E. Other **[SPECIFY]**...............................................

C. Bought from a restaurant

28. Who makes the decision on what you eat at home?

1. Mother 4. Respondent 7. Other **[SPECIFY]**.......................

2. Father 5. Family relation

3. Guardian 6 Friends

29. **CHECK SECTION D. EDUCATION, Q1:**

**🞏 ATTENDING SCHOOL → → → → → → → → → → → →**

**🞏** **NOT ATTENDING SCHOOL ↓**

**↓ ↓**

**CHECK SECTION F. EMPLOYMENT, Q1: ↓**

**🞏 NOT WORKING [GO TO Q30] ↓**

**🞏** **WORKING ↓**

**↓ ↓**

Where is the food you normally eat at school or work prepared? **[MULTIPLE RESPONSES]**

A. Prepared at home D. Bought from a chop-bar

B. Bought on the streets from food stands E. Bought at the school canteen

C. Bought from a restaurant F. Other **[SPECIFY]**...........................................

30. On the average, how many times in a day do you eat?

1. Once 3. Thrice 5. As and when I want

2. Twice 4. More than three 6. When food is available

7. Other **[SPECIFY]** ............................................

31. How many times do you eat meals with protein (eg. meat, beans, fish, eggs)?

1. Every meal 4. Once a week 7. As and when available

2. Once a day 5. Twice a week 8. Occasionally **[SPECIFY]**.......…………………....

3. Twice a day 6. Thrice a week 9. Other **[SPECIFY]** ...............................................

**H. HOPES AND ASPIRATIONS**

1. What do you want to become when you grow up?....................................................................................................

2. Why have you made this choice? **[PROBE TO SEE IF THIS IS THE RESPONDENT’S PERSONAL CHOICE OR IT IS DUE TO SOME OTHER INFLUENCE]**

1. Based on personal interest 4. Family relation’s influence

2. Parents’ decision 5. Family trade

3. Parents’ profession 6. Other **[SPECIFY]** .....................................

99. Don’t Know

3. What do you think you need to do to achieve that?

1. Study/learn 4. Pray/consult a god 7. Other **[SPECIFY]**......................

2. Travel overseas 5. Save money 99. Don’t Know

3. Travel to the urban centre 6. Work

4. What kind of help/assistance/support would you need to achieve that?

1. Financial support from family 4. Capacity building 7. Other **[SPECIFY]**.....................

2. NGO assistance 5. Own initiative/effort

3. District Assembly assistance 6. Friends 99. Don’t Know

5. Do you have any role-models?

1. Yes 2. No. **[GO TO SECTION I. ASSESSMENT BY CHILD ON PAGE 19]**

99. Don’t Know **[GO TO SECTION I. ASSESSMENT BY CHILD ON PAGE 19]**

6. Who is/are your role-model/s?............................................................................................................................

7. Why is/are she/he/they your role-model/s? ..............................................................................................

**I. ASSESSMENT BY CHILD**

1. Do you feel that you have enough food to eat ?

1. Yes 2. No

2. Explain your answer in question 1 .................................................................................................................................

3. How do you assess your standard of living?

1. High 3. Average 4. Other **[SPECIFY]** .............................

2. Low 99. Don’t Know

4. Give reasons for your assessment .......................................................................................................................

5. Do you have time for leisure? 1. Yes **[GO TO Q7]** 2. No

6. If No, why?............................................................................................................................................................

**[GO TO Q8]**

7. If Yes, how do you spend your leisure time? ......................................................................................................

8. Are you a happy person?

1. Yes 2. No 3. Some of the time 99. Don’t Know

9. Give reason(s) ..........................................................................................................................................................

10. **CHECK SECTION A. GENERAL INFORMATION Q4:**

**🞏 12 YEARS AND BELOW [END INTERVIEW]**

**🞏** **13 YEARS AND ABOVE [PROCEED WITH SECTIONS J, K, L & M]**

**J. INFLUENCE**

1. What is the most important thing in your life? ....................................................................................................

2. Why do you consider this the most important thing in your life? .......................................................................

3. What impact or how does this important thing affect your life?

..........................................................................................................................................................................

4. Who is the most important person in your life? .................................................................................................

5. Why do you consider this person the most important person in your life? ........................................................

6. What impact or how does this important person affect your life?

...........................................................................................................................................................................

7. Do you always agree with your parents?

1. Yes **[GO TO Q9]** 2. No 3. Sometimes

8. If No or Sometimes, what major issues do you normally disagree on?

...........................................................................................................................................................................

..................................................................................................................................................................................

9. Do you personally know of any children in your locality who have committed a crime?

1. Yes 2. No **[GO TO SECTION K. ADOLESCENT REPRODUCTIVE HEALTH ON PAGE 21]**

10. If Yes, what crime/s did they commit? ......................................................................................................................

................................................................................................................................................................................

...........................................................................................................................................................................

**K. ADOLESCENT REPRODUCTIVE HEALTH**

1. Where do you get information on sex education? **[MULTIPLE RESPONSES]**

A. Can’t remember I. Grandparents

B. Both parents J. Elder person

C. Father K. Friends

D. Mother L. Teacher

E. Siblings M. Pastor/Religious leader

F. Media (radio, TV, newspapers, internet) N. By observation

G. Books/Publications O. Other **[SPECIFY]**................................................

H. Cinema P. None

2. Are you married? 1. Yes 2. No

3. Are you betrothed? 1. Yes 2. No

4. How does one get pregnant? .........................................................................................................................…...

5. List the methods you know to prevent pregnancy ...........................................................................................…..

6. Do you believe in any of these? 1. Yes 2. No. 99. Don’t Know

7. What are your reasons for your answer in question 6? ........................................................................................

8. What is the ideal age for a child to have sex for the first time?.......................................years

9. Why is this the ideal age for a child to have sex for the first time? …………………………………………………….

…………………………………………………………………………………………………………………………………

10. What is the ideal age for a girl to have sex for the first time?....................................….years

11. Why is this the ideal age for a girl to have sex for the first time? ……………………………………………………..

…………………………………………………………………………………………………………………………………

12. What is the ideal age for a boy to have sex for the first time?.......................................years

13. Why is this the ideal age for a boy to have sex for the first time? …………………………………………………….

…………………………………………………………………………………………………………………………………

14. What are the factors that lead early sexual debut……………………………………………………………………….

………………………………………………………………………………………………………………………………..

15. What are the effects of early sexual debut………………..…………………………………………………………….

………………………………………………………………………………………………………………………………..

16. About how many unmarried young girls in their teens do you know of that have been pregnant?

1. Too many to count 3. A few 99. Don’t Know

2. None 4. Other **[SPECIFY]** ..........................................................................

17. Have you ever been pregnant or impregnated someone?

1. Yes 2. No **[GO TO Q17]**

18. What did you do about the pregnancy?......……...................................................................................................

19. Did you inform your parents about it?

1. Yes 2. No

20. What was their reaction?.................................…..................................................................................................

21. In your own opinion, how common is abortion in your area among teenage girls that get pregnant?

1. Not at all common 3. Very common 99. Don’t Know

2. Somewhat common 4. Other **[SPECIFY]** ...….....................................................

22. Where can such a girl go if she needs an abortion?

1. Hospital/Medical clinic 4. Traditionalist 7. Other **[SPECIFY]**...................................

2. Doctor 5. Self- induced 99. Don’t Know

3. Nurse 6. Friend

23. About how many unmarried young girls in their teens do you know of that have ever had an abortion?

1. A lot 3. None 99. Don’t Know

2. A few 4. Other **[SPECIFY]** .......................................................................

24. Have you ever had sexual intercourse? 1. Yes 2. No **[GO TO Q24]**

25. If Yes, how old were you when you had your first sexual intercourse?...............................................................

26. Have you ever been paid in cash or kind for sex? 1. Yes 2. No

27. If Yes, is that your regular source of income? 1. Yes 2. No

28. Are you aware of HIV infection/AIDS?

1. Yes 2. No **[GO TO Q29]**

29. If Yes, what are the modes of transmission? .....................................................................................................

30. How would you protect yourself against HIV infection/AIDS? Please state the methods that you know of.

(i)......................................................................................

(ii).....................................................................................

(iii)....................................................................................

(iv)....................................................................................

31. Do you believe in these? 1. Yes 2. No 99. Don’t Know

32. Give reasons .......................................................................................................................................................

33. Are you aware of Sexually Transmitted Diseases (STDs)?

1. Yes 2. No **[GO TO SECTION L. CHILDREN’S RIGHTS ON PAGE 22]**

34. If Yes, what types of STDs do you know of?

(i)......................................................................................

(ii).....................................................................................

(iii)....................................................................................

(iv)....................................................................................

35. How would you protect yourself against STDs? Please state the methods that you know of.

(i)......................................................................................

(ii).....................................................................................

(iii)....................................................................................

(iv)....................................................................................

36. Do you believe in any of these? 1. Yes 2. No 99. Don’t Know

37. Give reasons

(i)......................................................................................

(ii).....................................................................................

(iii)....................................................................................

(iv)....................................................................................

38. Are there laws that say one must be above a certain age before one can agree to have sexual intercourse?

1. Yes 2. No **[GO TO Q36]** 99. Don’t Know **[GO TO Q36]**

39. If Yes, what age is this? ..................................................

99. Don’t Know

40. Are there laws that say that one must be above a certain age before one can get married?

1. Yes 2. No **[GO TO Q38]** 99. Don’t Know **[GO TO Q38]**

41. If Yes, what age is this? ..................................................

99. Don’t Know

42. Are there laws that give a child the right to refuse betrothal or marriage?

1. Yes 2. No 99. Don’t Know

**L. CHILDREN’S RIGHTS**

1. Do children have fundamental rights?

1. Yes 2. No **[GO TO Q7]** 99. Don’t Know **[GO TO Q7]**

2. What are some rights children have?

(I)......................................................................................

(ii).....................................................................................

(iii)....................................................................................

(iv)....................................................................................

3. How did you get to know about these rights?

A. Can’t remember G. Books/Publications

B. Parents H. Cinema

C. Siblings I. Teacher

D. Grandparents J. Pastor/Religious Leader

E. Friends K. Other **[SPECIFY]**................................................

F. Media (radio, TV, newspapers)

4. Do you enjoy these rights?

1. Yes 2. No 99. Don’t Know

5. Explain your answer in question 4 ....................................................................................................................

6. How could the enjoyment of these rights be enhanced? ..................................................................................

7. Are you consulted in decisions that affect you at home?

1. Always 4. Once in a while/infrequently

2. Most of the time 5. Never **[GO TO Q9]**

3. Some of the time

8. If you are consulted, in what areas/issues are your opinions sought? **[MULTIPLE RESPONSES]**

A. Friends D. Academic subjects G. Clothing

B. Religion E. Leisure activities H. Hairstyle

C. TV programmes F. Work I. Other **[SPECIFY]** ..................................

9. Which of the underlisted do you have access to?

(I) Radio 1. Yes 2. No

(ii) Video 1. Yes 2. No

(iii) Cinema shows 1. Yes 2. No

(iv) Newspapers & other printed materials 1. Yes 2. No

(v) Television 1. Yes 2. No

(vi) Mobile Phone 1. Yes 2. No

(vii) Internet 1. Yes 2. No

10. Do you have friend/s who

(i) Smoke cigarettes? 1. Yes 2. No

(ii) Drink alcohol? 1. Yes 2. No

(iii) Take drugs? 1. Yes 2. No

11. If they take drugs, what type of drugs do they take? **[MULTIPLE RESPONSES]**

A. Marijuana (weed, pot, grass, Indian hemp)

B. Heroin (smack, horse)

C. Cocaine (crack, rock)

D. Inhalants (glue, gasoline)

E. Other **[SPECIFY]** ...........................................................................

12. Have you ever smoked any substance? 1. Yes 2. No **[GO TO Q17]**

13. What do you smoke?

1. Cigarette 4. Other [SPECIFY]………………………….

2. Shisha

3. Marijuana

14. How many times have you smoked?

1. Once 4. Can’t remember

2. Twice

3. Several times

15, What are your reasons for smoking?

1. Get high 4. Kill boredom/anxiety

2. For fun 5. Curiosity

3. Group culture 6. Other (SPECIFY)……………………………….

16. What are the factors that initiate children into smoking?

1. Friends smoke/group culture 4. Watched in Ads, movies, dramas etc.

2. Family member smokes 5. Other [SPECIFY]………………………….

3. Out of curiosity 6. Don’t Know

17. Have you ever taken alcohol 1. Yes 2. No **[GO TO Q22]**

18. First experience of alcohol

1. Friends 4. Drinking bar.

2. Elders in Family/household 5. Other [SPECIFY]………………………….

3. Party/ceremony

19. Ever got drunk? 1. Yes 2. No **[GO TO Q22]**

20. How often ever got drunk?

1. Never 4. Several times

2. Once 5. Can’t remember

3. Twice 6. Other [SPECIFY]………………………….

21. Reasons for drinking alcohol

1. Forgets worries 4. Taken as medicine 7.Other **[SPECIFY]**.....................

2. Boost confidence 5. Group culture/peer pressure

3. Whets appetite for food 6. Makes you prone to diseases 99. Don’t Know

22. What do you perceived as the effects of alcohol consumption?

1. Poor performance in school 4. Makes you forget worries 7.Other **[SPECIFY]**.....................

2. Become a deviant 5. Results in accidents and deaths

3. Makes your stronger/confident 6. As part of household/community ceremonial activities

99. Don’t Know

23. Do you know what Gambling is? 1. Yes 2. No

24. Do you perceive football betting as a game or gambling? 1. Game 2. Gambling99. Don’t Know

25. Have you ever Gambled? 1. Yes 2. No

26. Where do you play?

1. Online/phone 1. Yes 2. No
2. Casino 1. Yes 2. No
3. Sports bet facility 1. Yes 2. No

27. Where do you get money to play?

28. Does anyone in your family know you play any of these? 1. Yes 2. No 99. Don’t Know

29. Which of your family members know? (Father)

1. Mother 1. Yes 2. No
2. Father 1. Yes 2. No
3. Both parents 1. Yes 2. No
4. Siblings
5. Other family members

**M. COMPUTER AND INTERNET USE**

1. Have you ever used a computer?

1. Yes 2. No **[GO TO Q4]**

2. Have you used a computer in the last 12 months

1. Yes 2. No **[GO TO Q5]**

3. How often do you use computer?

4. What age did you first use the computer?

………………………………………………………………………………………………………………………………………..

5. Have ever used the Internet?

1. Yes 2. No **[GO TO SECTION N]** 99.

6. Where do you usually use the computer?

1. Home 2. School 3. Cafe 4. Public library 5. Other

7. Are you supervised using the internet?

| Place of usage | Who does supervision |
| --- | --- |
| Home 1. Yes 2. No |  |
| School 1. Yes 2. No |  |
| Café 1. Yes 2. No |  |
| Public library 1. Yes 2. No |  |
| Other (Specify) 1. Yes 2. No |  |

8. How often do you access the internet……………………………………………………………………………………………..

1. Once 2. Twice 3. Three times 4. Too many to count 99. Don’t Know

9. What age did you first use the internet?.....................................................................................................................

10. Did you receive some assistance the first time you used the internet?

1. Yes 2. No

11. How were you assisted with the use of the internet

12. What do you usually use the internet for?

1. Messaging/Chat

2. Schoolwork

3. Downloading music and videos

4. Browsing/surfing/social media

5. Research

6. Play games

13. What time of the day do you use the internet

……………………………………………………………………………………………………………………………………………..

……………………………………………………………………………………………………………………………………………..

14. How do you usually access the Internet

1. Phone

2. Computer/Laptop

3. Tablet

4. Other (Specify)

15. Have you ever seen pornography online in the last 12 months?

1. Yes 2. No**[GO TO Q19]**

16. How did you feel when you first came across it?

1. Shocked

2. Confused

3. Curious

4. Angry/disgusted

5. Excited

17. How did you first experience pornography?

| First experience | Response |
| --- | --- |
| Searched for it on my own | 1. Yes 2. No |
| Shown to them by someone’s without expecting it | 1. Yes 2. No |
| Came across it accidental online while doing something online | 1. Yes 2. No |
| Found it on a friend/family phone laptop | 1. Yes 2. No |
| Someone met online | 1. Yes 2. No |

18. Frequency of using internet for pornography in the last 12 months

1. Daily 2. Weekly 3 Monthly 4 As often as I want 5. Other (Specify)

19. How does Internet use benefit children?

…………………………………………………………………………………………………………………………………………………………………………………………………………………………………………………………………………………………………………

20. Have you ever pretended to be someone else online?

1. Yes 2. No

21. Why did you pretend to be someone else?

………………………………………………………………………………………………………………………………………………………………………………………………………………………………………………………………………………………………………………………………………………………………………………………………………………………………………………………………

22. Have you ever encountered violent contents online?

1. Yes 2. No

24 What was the violent content about?

………………………………………………………………………………………………………………………………………………………………………………………………………………………………………………………………………………………………………………………………………………………………………………………………………………………………………………………………

25 Who did you report about violent content?

………………………………………………………………………………………………………………………………………………………………………………………………………………………………………………………………………………………………………………………………………………………………………………………………………………………………………………………………

**N. TRADITIONAL PRACTICES**

1. List the common traditional practices in your locality? (Eg special rites for girls/boys, food taboos)

{i}........................................................................................................................................................................

...........................................................................................................................................................................

{ii}.......................................................................................................................................................................

...........................................................................................................................................................................

{iii}......................................................................................................................................................................

...........................................................................................................................................................................

{vi}......................................................................................................................................................................

2. What do you think of this/these practices?

{i}........................................................................................................................................................................

...........................................................................................................................................................................

{ii}.......................................................................................................................................................................

...........................................................................................................................................................................

{iii}......................................................................................................................................................................

...........................................................................................................................................................................

{vi}......................................................................................................................................................................

3. Give reasons for your opinion ...........................................................................................................................

{i}........................................................................................................................................................................

...........................................................................................................................................................................

{ii}.......................................................................................................................................................................

...........................................................................................................................................................................

{iii}......................................................................................................................................................................

...........................................................................................................................................................................

{vi}......................................................................................................................................................................

**DEPARTMENT OF CHILDREN – MINISTRY OF GENDER CHILDREN AND SOCIAL PROTECTION**

**SITUATIONAL ANALYSIS OF CHILDREN IN GHANA 2018**

***INTERVIEW GUIDE***

**Children**

1. Kindly tell me about yourself:

**Probe:** Age, educational level, etc.

1. Is smoking common among children in your community/locality?
2. What kind of substance do children smoke?

**Probe:** The circumstances under which a substance is used if mentioned a child

1. Where do children usually smoke and reasons for choosing the place of smoking?
2. Why do children smoke in your community/locality?
3. What environmental factors influence children to initiate smoking?
4. What are the perceived effects of smoking on children?

**Key informants**

1. Kindly tell me about yourself:

**Probe:** Age, educational level, etc.

1. What is your current position at your organisation?

**Probe:** How long has you acted in this position

1. What are your duties and responsibilities?
2. Is smoking common among children in your community/locality?
3. What kind of substance do children smoke?

**Probe:** The circumstances under which a substance is used if mentioned a child

1. Where do children usually smoke and reasons for choosing the place of smoking?
2. Why do children smoke in your community/locality?
3. What regulations are in place to control childhood smoking?

**Probe:** What laws and conventions on childhood smoking has Ghana signed or ratified?
